# Identification of Prognostic lncRNA Related to the Immune Microenvironment of Soft Tissue Sarcoma

**DOI:** 10.1155/2022/9471558

**Published:** 2022-02-02

**Authors:** Wang-Ying Dai, Bin Wang, Jian-Yi Li, Zong-Ping Luo

**Affiliations:** ^1^Orthopaedic Institute, Department of Orthopaedics, The First Affiliated Hospital of Soochow University, 708 Renmin Rd., Suzhou, Jiangsu 215007, China; ^2^Department of Medical Oncology, The First Affiliated Hospital of Wenzhou Medical University, Wenzhou 325000, China; ^3^Department of Orthopaedic Surgery, The Affiliated Hospital of Qingdao University, Qingdao 266000, China

## Abstract

**Background:**

Soft tissue sarcoma is a malignant tumor with high degree of malignancy and poor prognosis, originating from mesenchymal tissue. Long noncoding RNAs (lncRNAs) are involved in various biological and pathological processes in the body. They perform preprocessing, splicing, transport, degradation, and translation of mRNA to achieve posttranscriptional level regulation, resulting in the occurrence, invasion, and metastasis of tumors. Therefore, they are highly relevant with regard to early diagnoses and as prognostic indicators.

**Objective:**

The objective of the present study was to identify immune microenvironment-related lncRNAs that can be used to predict soft tissue sarcomas.

**Methods:**

Clinical data and follow-up data were obtained from the cBioPortal database, and RNA sequencing data used for the model structure can be accessed from The Cancer Genome Atlas (TCGA) database. LncRNAs were screened by differential expression analysis and coexpression analysis. The Cox regression model and Kaplan–Meier analysis were used to study the association between lncRNAs and soft tissue sarcoma prognosis in the immune microenvironment. Unsupervised cluster analysis was then completed to discover the impact of screening lncRNAs on disease. We constructed an mRNA-lncRNA network by Cytoscape software. Finally, qRT-PCR was used to verify the difference in the expression of the lncRNAs in normal cells and sarcoma cells.

**Results:**

Unsupervised cluster analysis revealed that the 210 lncRNAs screened showed strong correlation with the tumor immune microenvironment. Two signatures containing seven and five lncRNAs related to the tumor microenvironment were constructed and used to predict overall survival (OS) and disease-free survival (DFS). The Kaplan–Meier (K-M) survival curve showed that the prognoses of patients in the high-risk and low-risk groups differed significantly, and the prognosis associated with the low-risk group was better than that associated with the high-risk group. Two nomograms with predictive capabilities were established. qRT-PCR results showed that the expression of AC108134.3 and AL031717.1 was significantly different in normal and sarcoma cells.

**Conclusion:**

In summary, the experimental results showed that lncrnA associated with immune microenvironment was related to tumor, which may provide a new idea for immunotherapy of STS.

## 1. Introduction

Soft tissue sarcoma is a heterogeneous malignant mesenchymal tumor [1]. It accounts for more than 20% of solid malignant tumors in children and less than 1% of solid malignant tumors in adults [2]. The incidence of the disease is relatively low, but it is highly malignant in most patients and is associated with a poor prognosis [3]. Therefore, prognostic indicators of the disease and early diagnosis are vitally important.

Previous studies have revealed that the tumor immune microenvironment plays an important part in the occurrence and development of tumors [4–6]. The tumor microenvironment (TME) can affect the biological characteristics of tumor cells by regulating the expression of long noncoding RNAs (lncRNAs) [7]. And lncRNAs can also regulate TME [8–10]. Studies have shown that the stimulation of interleukin 6 (IL-6) can cause the spread of liver cancer cells, which are mainly caused by the promotion of lncTCF7 expression through the transcription (STAT) signaling pathway [11]. However, the abnormal regulation of a variety of oncogenes and tumor suppressor genes can lead to tumorigenesis [12], and lncRNAs can participate in malignant changes in cells and tumorigenesis by regulating important oncogenes or suppressor genes [13]. For example, lncRNA RUSC1-AS1 plays an important role in the occurrence of liver cancer, mainly by regulating the PI3K/AKT signaling pathway [14]. lncRNA KCNQ1OT1 can promote the growth of osteosarcoma through enhanced aerobic glycolysis [15]. Therefore, lncRNA related to the tumor immune microenvironment has the possibility of being a prognostic indicator. Moreover, research into such markers can provide the theoretical basis for the development of new therapeutic targets and strategies and can guide first-line treatment [16].

In the present study, RNA sequencing data and clinical data were collected and sorted out, and the osteosarcoma immune score is quantified based on the ESTIMATE algorithm [17]. Differential expression analysis and immune-related mRNA coexpression analysis were used to identify immune-related lncRNAs associated with the TME. Finally, a series of bioinformatic methods were used to determine the prognostic value of the identified lncRNAs.

## 2. Materials and Methods

### 2.1. Data Collection and Pretreatment

Clinical data and follow-up data were downloaded from the cBioPortal database (http://www.cbioportal.org/) [18]. RNA sequencing data were obtained from TCGA data portal (https://cancergenome.nih.gov/) [19]. The collected samples only retained the specimens of the tumor at the primary site (259 cases). All data from this study are available to the public.

### 2.2. Differences in Tumor Microenvironmental Immune Score and Prognosis

The ESTIMATE, an algorithm inferring tumor purity, stromal score, and immune cell admixture from expression data, was used in the *R* language software to evaluate matrix score and immune score on the samples by executing ssGSEA [17, 20]. The scores were sorted, and X-tile software [21] was used to divide the samples into high-score and low-score groups based on the median score. The prognostic differences between the two groups were then compared using K-M survival curves (including OS and DFS).

### 2.3. Identification of Immune Microenvironment-Related lncRNAs in Soft Tissue Sarcomas

In order to understand the reasons for the differences between the high-score and low-score groups, we analyzed the differences in immune scores between the high-score and low-score of lncRNA in the microenvironment. After obtaining the lncRNA expression data, the “limma” software package—written in the *R* programming language—was used to compare lncRNA expression in the high-score and low-score groups and to perform differential expression analysis [22]. When ∣log2FC | >1 and FDR < 0.05, the lncRNA expression between the high-score and low-score groups is considered significantly different. Immune-related mRNA data were downloaded from the ImmPort database (https://www.immport.org/) [23], and we identify immune-related lncRNAs by Pearson correlation analysis (correlation coefficient | *r* | ≥0.4 and *p* < 0.05). Finally, the results obtained using the two methods described above were combined to identify the immune microenvironment-related lncRNAs of soft tissue sarcomas.

### 2.4. Unsupervised Cluster Analysis

To determine the correlation between the screened lncRNAs and immunity, the “Consensus Cluster Plus” software package was used to perform unsupervised cluster analysis [24]. The K-M survival curve was used after the subgroups were divided, and the log-rank test was used to determine the differences in OS and DFS between the groups. The differences in the microenvironment scores between the groups were then compared.

### 2.5. Independent Prognostic Analysis and Clinical Correlation Analysis

First, we performed a single-factor Cox analysis (*p* value <0.05) to identify the lncRNAs related to prognosis. LASSO regression analysis was performed to avoid overfitting [25]. Multifactor Cox analysis was then carried out. The most appropriate differentially expressed lncRNAs related to OS or DFS and associated with the immune microenvironment were selected. The corresponding lncRNA-derived risk score for each patient with soft tissue sarcoma was simultaneously calculated using the following formula: score = ∑_*i*=0_^*n*^PSI × *β*_*i*_ (where *β* is the regression coefficient).

The patients were then divided into high-risk and low-risk groups. K-M survival analysis was used to compare the prognostic differences between the high-risk and low-risk groups. The receiver operating characteristic (ROC) curves for 3, 5, and 7 years were used simultaneously to verify the prediction efficiency of the signatures [20, 26, 27]. And the area under the curve (AUC) was used to represent the differentiation of the nomogram. Finally, combined with the clinical data, single- and multifactor Cox analyses were performed to determine the independent predictors of lncRNA prognosis in the TME.

### 2.6. Construction of the Nomogram

We developed a nomogram to predict the OS and DFS of lncRNAs in the tumor immune microenvironment. Firstly, the univariate COX analysis was performed to filter prognostic variables, which will be further included in the multivariate COX analysis. Secondly, based on independent prognostic variables, two nomograms were established for predicting the OS and DFS, respectively. The time-dependent ROC curves were used to create the nomogram prognostic prediction chart [26, 27]. Simultaneously, the 3-, 5-, and 7-year survival rate calibration curves were used to correct the nomogram prognostic prediction chart.

### 2.7. Construction of the mRNA-lncRNA Network

A regulatory mRNA-lncRNA network was constructed using Cytoscape (version 3.7.2), and the interaction between mRNA and lncRNA was analyzed using the Pearson test (∣*r* | >0.4, *p* < 0.05) [28].

### 2.8. Cell Culture

Normal human dermal fibroblast cells (HDF-a) and human fibrosarcoma cells (HT1080) were purchased from the Cell Storage Center of otwo. All cells are cultured in Dulbecco's Modified Eagle's Medium (DMED) containing 10% FBS and 1% streptomycin/penicillin. Then, place the cells in a 37°C, 5% CO_2_ incubator for culture. Change the medium once a day. The RNA is extracted when the cells grow to 80% confluent.

### 2.9. Quantitative Real-Time PCR (qRT-PCR)

Use TRIzol (ThermoFisher Scientific, USA) to extract total cell RNA. Follow the steps of PrimeScrip reverse transcription kit (Takara, Japan) to reverse transcription into cDNA. Configure the PCR reaction system and analyze it according to the SYBR Premix Ex Taq (Takara, Japan) instruction. The relative expression is expressed by 2^−∆∆Ct^. Repeat the experiment 3 times independently for each sample (Table 1).

### 2.10. Statistical Analyses

All analyses were performed using *R* version 4.0.5. Unless otherwise noted, statistical significance was set at *p* < 0.05.

## 3. Results

### 3.1. Relationship between TME Immune Score and Patient Prognosis

**Figure 1:**
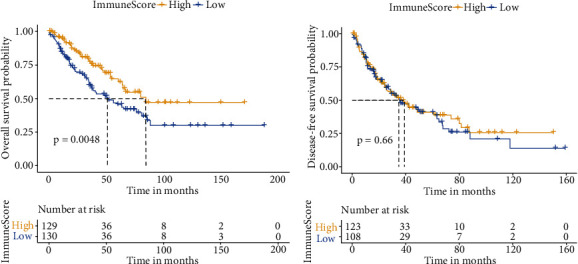
Correlation between the tumor microenvironmental immune score and the prognosis of soft tissue sarcoma. (a) K-M analysis of overall survival (OS) in the high and low immune score groups.(b) K-M analysis of disease-free survival (DFS) in the high and low immune score groups.

Excluding nonprimary tumor specimens, we included 259 cases in the analysis. Of these, there were 129 cases in the high immune score group and 130 cases in the low immune score group. K-M survival analysis revealed that OS differed significantly whereas DFS did not (Figure 1).

### 3.2. Overview of lncRNAs Related to the Immune Microenvironment

**Figure 2:**
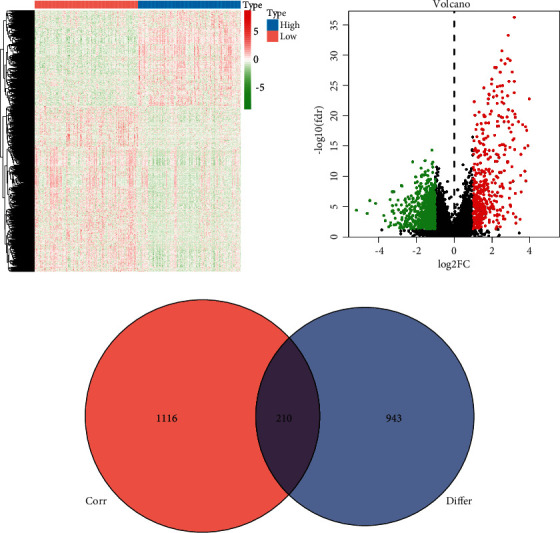
Screening of lncRNAs related to the tumor immune microenvironment. (a) lncRNA heat map obtained by differential analysis of the immune scores (Supplementary document 2). (b) lncRNA volcano map obtained by differential analysis of the immune scores. (c) Venn diagram of the lncRNA data obtained by differential analysis and the lncRNA data obtained by coexpression analysis.

To identify the lncRNAs that were differentially expressed in the high and low immune score groups, we first screened and obtained 1153 differentially expressed lncRNAs according to the conditions and methods described above (Figure 2(a)). The volcano map reveals that the number of upregulated (red) lncRNAs is the majority, reflecting that most of the immune-related lncRNAs promote the occurrence and development of tumors (Figure 2(b)). The obtained immune-related mRNAs and all the lncRNAs were then coexpressed, and 1326 immune-related lncRNAs were identified. Finally, the intersection of lncRNAs obtained by two methods was used to identify the 210 immune-related lncRNAs (Figure 2(c)).

### 3.3. LncRNA-Based Clusters Significantly Associated with Prognosis and Immune Microenvironment Scores

**Figure 3:**
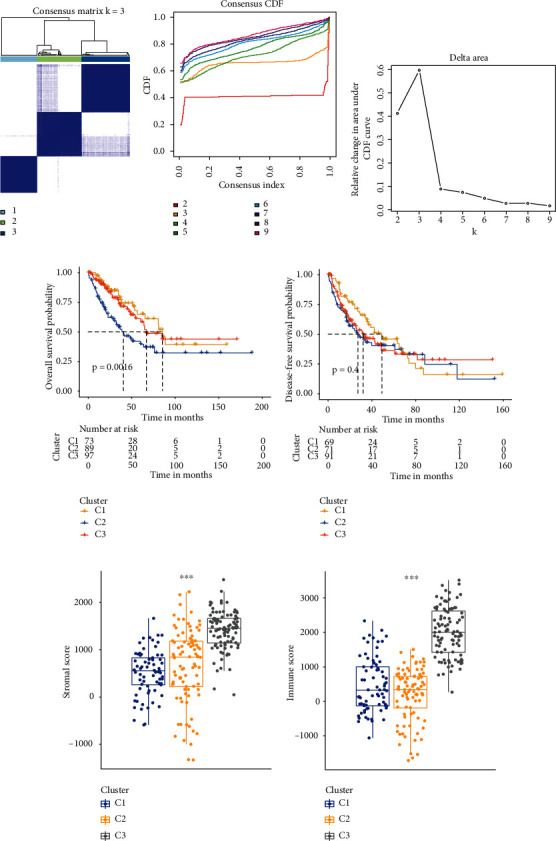
LncRNA-based clusters significantly associated with prognosis and immune microenvironment scores. (a) Consensus clustering matrix for *k* = 3. (b) Cumulative distribution function (CDF) shows the cumulative distribution function when *k* takes different values. (c) The delta area plot shows the relative change of the area under the CDF curve between *k* and *k* − 1; K-M survival curve of overall survival (OS) (d) and disease-free survival (DFS) (e). Comparison of stromal (f) and immune score among three clusters (g).

Based on the consensus matrix heat map, the 258 samples were clearly divided into three clusters (Figure 3(a)). In addition, by comprehensively analyzing the relative change in area under the cumulative distribution function, three clusters were determined (Figures 3(b) and 3(c)). K-M survival analysis subsequently revealed that OS differed significantly but DFS did not (Figures 3(d) and 3(e)). Finally, the differences in immune scores between the three clusters were compared, and it was found that the third cluster was significantly higher than the first two clusters in both the matrix and immune scores (Figures 3(f) and 3(g)).

### 3.4. Construction of lncRNA Prognostic Model Related to the Immune Microenvironment

**Figure 4:**
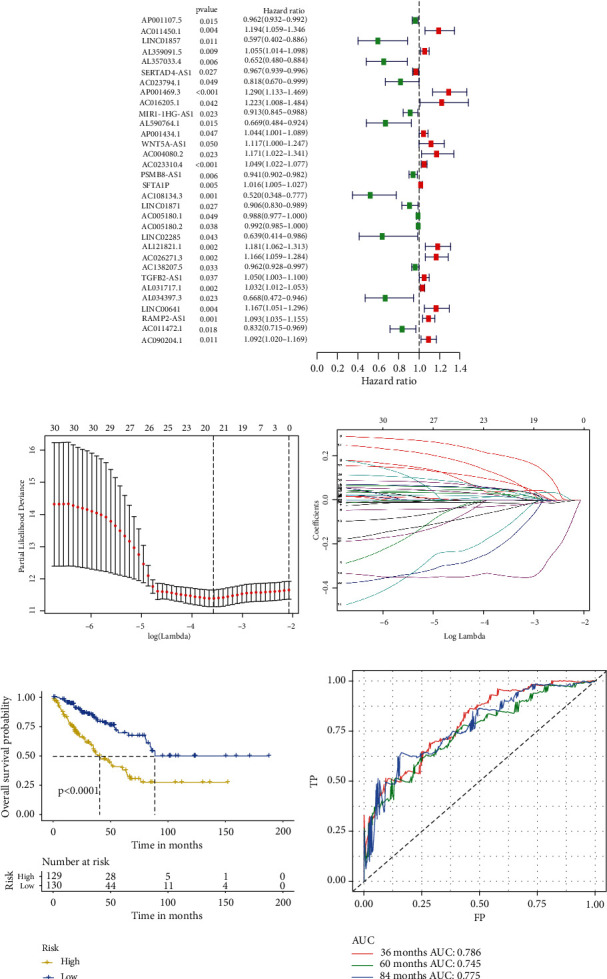
Establishment of the Cox regression model of overall survival (OS). (a) Univariate Cox analysis of OS-related variables (Supplementary document 3). (b, c) Least absolute shrinkage and selection operator (LASSO) regression was used to construct a predictive model to select the optimal lncRNA related to OS. (d) Survival analysis of the prognostic model. The upper part shows the K-M curve of the high-risk and low-risk groups; the bottom part shows the change in the number of surviving patients in the high-risk and low-risk groups over time. Yellow represents high-risk groups, and blue represents low-risk groups. (e) Receiver operating characteristic (ROC) curve of the forecast model at 3, 5, and 7 years. Red represents 3 years, green represents 5 years, and blue represents 7 years.

**Figure 5:**
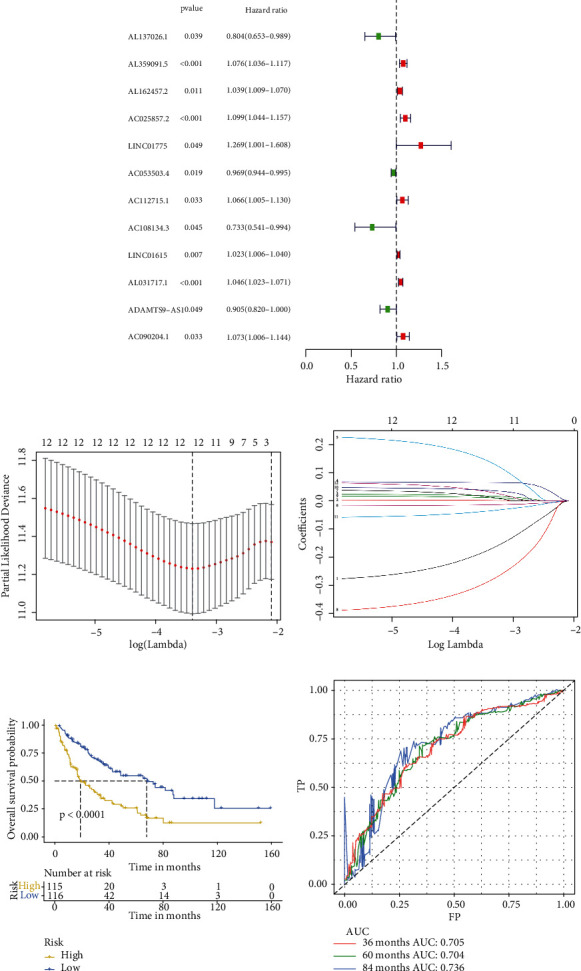
Establishment of the Cox regression model of disease-free survival (DFS). (a) Univariate Cox analysis of overall survival- (OS-) related variables (Supplementary document 3). (b, c) Least absolute shrinkage and selection operator (LASSO) regression was used to construct a predictive model to select the optimal lncRNA related to DFS. (d) Survival analysis of the prognostic model. The upper part shows the K-M curve of the high- and low-risk groups; the bottom part shows the change in the number of surviving patients in the high- and low-risk groups over time. Yellow represents high-risk groups, and blue represents low-risk groups. (e) The receiver operating characteristic (ROC) curve of the forecast model at 3, 5, and 7 years. Red represents 3 years, green represents 5 years, and blue represents 7 years.

**Figure 6:**
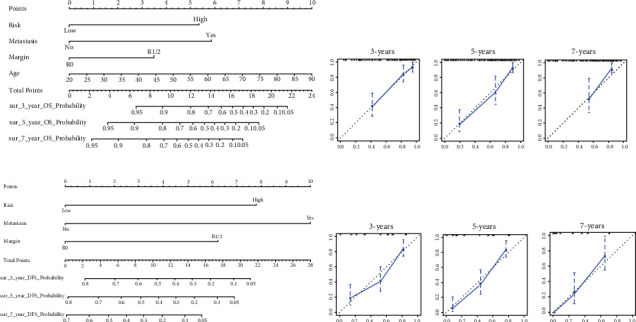
Overall survival (OS) and disease-free survival (DFS) nomograms based on lncRNAs related to the immune microenvironment of patients with osteosarcoma. (a) Nomogram predicts the OS of patients with osteosarcoma; calibration chart of RNA nomogram: observe the consistency between the nomogram forecasts and the observed OS values for 3 (b), 5 (c), and 7 (d) years. (e) The nomogram predicts the DFS of patients with osteosarcoma; calibration chart of RNA nomogram: observe the consistency between the nomogram forecasts and observed values of DFS after 3 (f), 5 (g), and 7 (h) years.

First, single-factor Cox regression analysis identified 32 and 12 lncRNAs correlated with OS (Figure 4(a)) and DFS (Figure 5(a)), respectively. LASSO regression analysis was used to reduce overfitting (Figures 4(b) and 4(c) and Figures 5(b) and 5(c)), and 20 and 12 lncRNAs were determined to be extremely relevant to the prognosis of OS and DFS, respectively. And multifactor Cox regression analysis was used to screen 12 and 8 lncRNAs related with independent prognoses of OS (Table 2) and DFS (Table 3), respectively. K-M survival analysis revealed significant differences between the low-risk and high-risk groups (Figures 4(d) and 5(d)). Simultaneously, the ROC curve AUC value of the model was greater than 0.7, indicating that the model was more accurate (Figures 4(e) and 5(e)). Then, combined with clinical indicators for analysis, the results indicated that lncRNA-derived risk indicators could be used as an independent prognostic factor for OS and DFS models (Tables 4–7). Finally, two nomograms were established based on independent prognostic predictors. The calibration curves of the 3-, 5-, and 7-year survival rates revealed good agreement between the predicted results and the actual results (Figure 6).

### 3.5. Regulatory Network of mRNA and lncRNA

**Figure 7:**
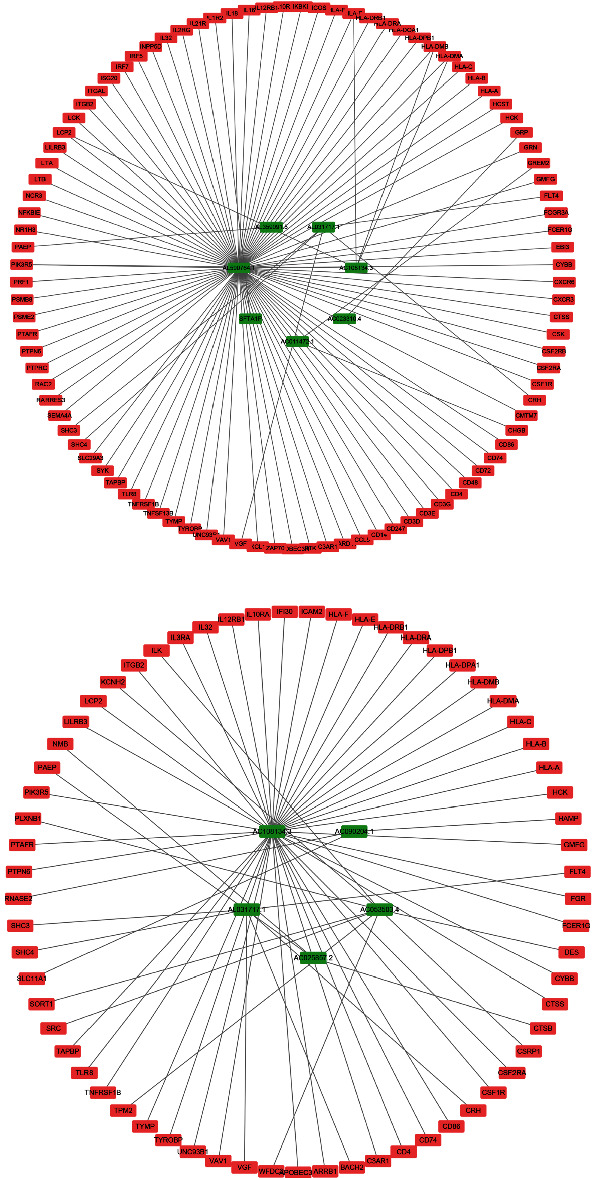
mRNA-lncRNA regulatory network. (a) The final overall survival (OS) signature. (b) The final disease-free survival (DFS) signature.

The construction of mRNA-lncRNA coexpression network helps us to further understand the regulatory relationship between mRNA and lncRNA (Supplementary document 1). We obtained 7 lncRNAs and 97 mRNAs that were incorporated into the final OS signature, 102 of which were related to network generation (Figure 7(a)). At the same time, 5 lncRNAs and 65 mRNAs were selected for incorporation into the final DFS signature, and 65 associations were used to generate another network (Figure 7(b)). Two lncRNAs—AC108134.3 and AL031717.1—were concurrently combined into OS and DFS signatures. Each had five RNAs as targets.

### 3.6. Expression of AC108134.3 and AL031717.1 mRNA in Fibroblast and Fibrosarcoma Cells

**Figure 8:**
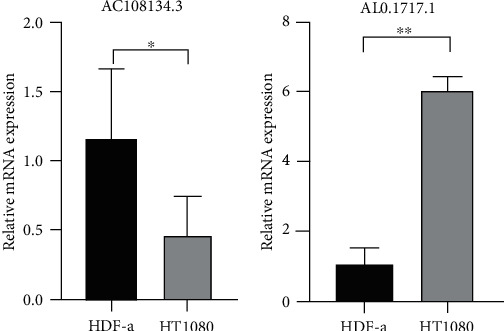
Expression of AC108134.3 and AL031717.1 in fibroblast and fibrosarcoma cells. (a) AC108134.3. (b) AL031717.1. Compared to the normal group, ^∗^*p* < 0.05, ^∗∗^*p* < 0.01.

By qRT-PCR analysis of the two lncRNAs, it can be found that the expression of AC108134.3 (Figure 8(a)) in normal fibroblasts is significantly higher than that in fibrosarcoma cells, while the expression of AL031717.1 is the opposite (Figure 8(b)).

## 4. Discussion

In recent years, many studies have targeted the relationship between lncRNA and tumors [29–31]. However, research on lncRNAs in soft tissue sarcomas remains insufficient. In this study, lncRNAs related to the immune microenvironment were jointly screened from the differences between the sample groups and the coexpression of immune-related mRNAs in the database. The prediction model based on the screened lncRNAs and clinicopathological data performed well.

In the present study, the samples were divided into high-score and low-score groups according to their immune microenvironment scores to compare different lncRNAs. The results are presented as a volcano map. There are numerous positive correlations, indicating that most immune-related lncRNAs in the immune microenvironment promote the formation and development of tumors. We then downloaded immune-related mRNA data and all the lncRNA data from the ImmPort database for coexpression analysis. The two results described above were then combined and screened to obtain 210 immune-related lncRNAs. Subsequently, an unsupervised cluster analysis was performed, in which the lncRNAs were divided into three groups. The results showed that OS differed significantly among the three groups. The survival rates of the first and third clusters were higher than that of the second cluster. Furthermore, the matrix and immune scores of the third cluster were both the highest, indicating that these lncRNAs are closely related to immunity. There were also differences in the levels of immune cells between the third cluster and the other two clusters, indicating that lncRNAs related to the immune microenvironment may influence the prognosis of patients.

Indepth studies show that lncRNAs have roles in epigenetic modification and transcriptional and posttranscriptional regulation. Different lncRNAs are related to the occurrence and development of tumors, and they are usually expressed abnormally in cancers. LncRNA not only participates in tumor formation but also inhibits the occurrence and development of tumors. Studies have shown that the expression of lncRNAs can be used as a biomarker for cancer diagnosis [32], may be related to the prognosis of tumors, and can be used as a potential biomarker to guide prognosis [33]. In the regulatory network constructed in the present study, lncRNA SFTA1P has been reported to downregulate miR-4766-5p through the PI3K/AKT/mTOR signaling pathway to promote liver cancer growth [34]. Our research has identified lncRNAs contained in two unreported signatures, i.e., AC108134.3 and AL031717.1. In addition, we performed qRT-PCR analysis on these two lncRNA. It is found that these two lncRNAs are significantly different in normal cells and tumor cells, which verifies the correctness of our results. This study provides a theoretical basis for further study of these two lncRNAs as prognostic biomarker. In addition to the lncRNAs, the corresponding targeted mRNAs are also involved in immune regulation. For example, previous studies have shown that SHC3 is functionally relevant to TRIP13-mediated tumor growth and metastasis [35, 36].

Undeniably, this study still has several limitations that need to be improved. First, the data is downloaded from the public TCGA database; so, a certain degree of selection bias cannot be ruled out, and the clinical data were not comprehensive. Second, our data were based on theoretical analysis, and further basic experiments are needed to verify the differences and specific mechanisms of these lncRNAs.

## 5. Conclusion

The experimental results showed that lncRNA associated with immune microenvironment was related to tumor, which may provide a new idea for immunotherapy of STS.

## Figures and Tables

**Table 1:** qRT-PCR primer sequence.

	Primer sequences (5′–3′)	
AC108134.3	Forward:	CAGAACTGGAAGACTCCAG
	Reverse:	GTTAAACGCTTACCAGCAC
AL031717.1	Forward:	GTGAAACGTCTGGAACAGGC
	Reverse:	CAGTCACATCGTCTTCACCC
GAPDH	Forward:	TGGAGAAACCTGCCAAGTATG
	Reverse:	GGAGACAACCTGGTCCTCAG

**Table 2:** Multivariate COX analysis of OS-related lncRNA.

ID	Coef	HR	HR.95L	HR.95H	*p* value
AL359091.5	0.088060753	1.092054465	1.046274344	1.139837712	5.57*E*-05
SERTAD4-AS1	-0.039187094	0.961570789	0.925658604	0.998876235	0.043605576
AP001469.3	0.274565283	1.31595848	1.138002144	1.521742934	0.000212342
AL590764.1	-0.558159305	0.572261454	0.355597044	0.920938959	0.021491453
AC004080.2	0.181422327	1.19892141	1.020289712	1.408827836	0.02752551
AC023310.4	0.060803514	1.06269009	1.034990628	1.091130872	6.42*E*-06
SFTA1P	0.020742614	1.020959237	1.010411081	1.031617511	9.05*E*-05
AL031717.1	0.03709739	1.037794087	1.016806929	1.059214425	0.00037236
RAMP2-AS1	0.085356525	1.089105291	1.022294007	1.160282978	0.008227374
AC011472.1	-0.136966991	0.871999009	0.748569258	1.015780789	0.07859557
AC090204.1	0.136071208	1.145763478	1.065674907	1.231870938	0.00023284
AC108134.3	-0.372794064	0.688807068	0.416602019	1.138869125	0.146195493

OS: overall survival; HR: hazard ratio.

**Table 3:** Multivariate COX analysis of DFS-related lncRNA.

ID	Coef	HR	HR.95L	HR.95H	*p* value
AL137026.1	-0.260990123	0.770288528	0.616787718	0.961991297	0.021351877
AC025857.2	0.095566057	1.100281501	1.046109338	1.157258937	0.000207341
LINC01775	0.220716396	1.246969735	0.963303155	1.614168408	0.093727035
AC053503.4	-0.0267261	0.973627881	0.948107598	0.999835096	0.048594386
AC108134.3	-0.397730261	0.671843226	0.485753745	0.929222522	0.01623539
LINC01615	0.023375494	1.023650842	1.00480508	1.042850068	0.013679342
AL031717.1	0.047251259	1.048385393	1.024295589	1.07304175	6.78*E*-05
AC090204.1	0.061719051	1.063663467	0.988832025	1.144157898	0.097272497

DFS: disease-free survival; HR: hazard ratio.

**Table 4:** Univariate Cox analysis of clinicopathological factors in patients with OS-related soft tissue sarcoma.

ID	HR	HR.95L	HR.95H	*p* value
Risk	2.819425565	1.844548576	4.30954252	1.68*E*-06
Age	1.020203157	1.004851698	1.035789145	0.009720255
Type 1	0.822698599	0.499921639	1.353878154	0.442550772
Type 2	0.674303076	0.359714697	1.264014624	0.219006691
Type 3	0.895366536	0.477370431	1.679369275	0.730530813
Metastasis	3.013737497	1.833539793	4.953595082	1.35*E*-05
Race 1	1.085399289	0.132018532	8.923683664	0.939229846
Race 2	0.791066954	0.108401374	5.772868961	0.817221062
Postoperative_radiotherapy	0.988244054	0.618585136	1.578806623	0.960542537
Margin	2.553571993	1.667915977	3.909507441	1.60*E*-05
Sex	0.854557794	0.571819232	1.277097695	0.443231109
Tumor tissue site 1	1.222885503	0.726840069	2.057466308	0.448437108
Tumor tissue site 2	1.224341377	0.756895753	1.980473268	0.409450534

Type 1: dedifferentiated liposarcoma; type 2: leiomyosarcoma; type 3: undifferentiated pleomorphic sarcoma/malignant fibrous histiocytoma/high-grade spindle cell sarcoma; race 1: black; race 2: white; tumor tissue site 1: extremity; tumor tissue site 2: retroperitoneum/upper abdominal; OS: overall survival; HR: hazard ratio.

**Table 5:** Multivariate Cox analysis of clinicopathological factors in patients with OS-related soft tissue sarcoma.

ID	HR	HR.95L	HR.95H	*p* value
Risk high	4.292313248	2.360476228	7.805184735	1.80*E*-06
Metastasis Y	4.916605917	2.762421043	8.750662324	6.15*E*-08
Margin R1/2	2.582072435	1.466700775	4.545642964	0.001011618
Age	1.039640904	1.018696793	1.06101562	0.000181136

OS: overall survival; HR: hazard ratio.

**Table 6:** Univariate Cox analysis of clinicopathological factors in patients with DFS-related soft tissue sarcoma.

ID	HR	HR.95L	HR.95H	*p* value
Risk	2.509702365	1.743348261	3.612936155	7.43*E*-07
Age	1.010257968	0.99759091	1.023085868	0.112898635
Type 1	0.805277923	0.515808804	1.257195552	0.340646916
Type 2	0.728409377	0.424318464	1.250429256	0.250402178
Type 3	0.80790002	0.45612612	1.430969228	0.464561685
Metastasis	4.937153189	3.140400876	7.761901289	4.60E-12
Race 1	2.121508392	0.264322128	17.02769987	0.479070219
Race 2	1.913142406	0.265587372	13.78120443	0.519606531
Postoperative_radiotherapy	1.169024353	0.783807692	1.743562804	0.443870378
Margin	2.084657548	1.422072136	3.055961075	0.000167028
Sex	1.089657382	0.766928708	1.548192416	0.631832584
Tumor tissue site 1	0.914504601	0.578235491	1.446328837	0.702367293
Tumor tissue site 2	1.006297116	0.66911367	1.513395902	0.975947716

Type 1: dedifferentiated liposarcoma; type 2: leiomyosarcoma; type 3: undifferentiated peomorphic sarcoma/malignant fibrous histiocytoma/high-grade spindle cell sarcoma; race 1: black; race 2: white; tumor tissue site 1: extremity; tumor tissue ste 2: retroperitoneum/upper abdominal; DFS: disease-free survival; HR: hazard ratio.

**Table 7:** Multivariate Cox analysis of clinicopathological factors in patients with DFS-related soft tissue sarcoma.

ID	HR	HR.95L	HR.95H	*p* value
Risk high	2.922715806	1.752909261	4.873194452	3.93*E*-05
Metastasis Y	3.962575012	2.428430892	6.465903879	3.56*E*-08
Margin R1/2	2.356620559	1.421957355	3.905644879	0.000881889

DFS: disease-free survival; HR: hazard ratio.

## Data Availability

This study was carried out using publicly available data from the cBioPortal database at http://www.cbioportal.org/ and the TCGA data portal at https://cancergenome.nih.gov/. And you can contact us for analysis code.

## Abbreviations

TME: Tumor microenvironment
TCGA: The Cancer Genome Atlas
lncRNAs: Long noncoding RNAs
ROC: Receiver operating characteristic
OS: Overall survival
DFS: Disease-free survival
K-M: Kaplan–Meier
CDF: Cumulative distribution function
DMED: Dulbecco's Modified Eagle's Medium
qRT-PCR: Quantitative real-time PCR.

## Supplementary Materials

Supplementary 1 Supplementary document 1: raw data of mRNA-lncRNA coexpression network.

Supplementary 2 Supplementary document 2: raw data of lncRNA heat map.

Supplementary 3 Supplementary document 3-1: raw data of univariate Cox analysis of overall survival- (OS-) related variables.

Supplementary 4 Supplementary document 3-2: raw data of univariate Cox analysis of OS-related variables.
